# Penile Angiokeratoma (Peaker): A Distinctive Subtype of Genital Angiokeratoma

**DOI:** 10.7759/cureus.3793

**Published:** 2018-12-28

**Authors:** Pallavi Basu, Philip R Cohen

**Affiliations:** 1 Dermatology, University of California, San Diego, USA; 2 Dermatology, San Diego Family Dermatology, San Diego, USA

**Keywords:** angiokeratoma, genital, peaker, penile, penis, rejuvenation, scrotal, scrotum, vagina, vaginal

## Abstract

Penile angiokeratomas (peakers) are uncommon, benign vascular tumors typically presenting as multiple lesions on the corona of the glans penis. They have been observed in 21 men. They range from 0.5 to 5 millimeters in size and initially appear in both young and old men. They are usually asymptomatic and are managed conservatively. They are rarely associated with systemic diseases. Symptomatic or cosmetically undesirable lesions can be treated with cryotherapy, electrodessication, excision, laser therapy, or sclerotherapy. We present a man with a solitary angiokeratoma of the glans penis and discuss the unique features of penile angiokeratomas.

## Introduction

Genital angiokeratomas are benign, usually idiopathic, vascular lesions occurring most commonly on the scrotum or vulva [1-3]. Angiokeratomas of the penis are rare and can develop as solitary or multiple lesions on the glans penis [4-16]. They are morphologically and pathologically similar to angiokeratomas in other locations. A man with an angiokeratoma on his glans penis is described and the distinctive features of penile angiokeratomas (peakers) are reviewed.

## Case presentation

**Figure 1:**
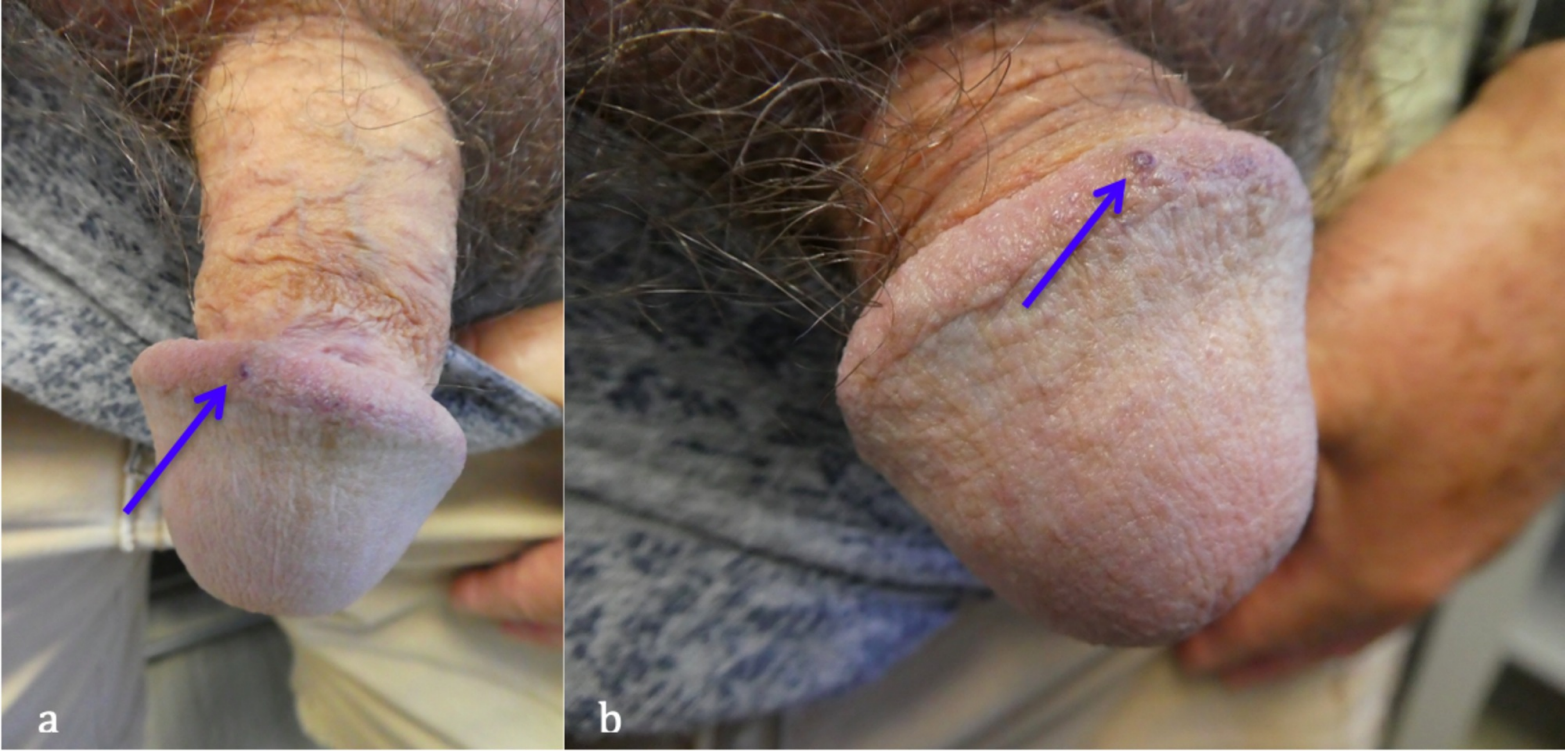
Distant (a) and closer (b) views of a solitary penile angiokeratoma or “peaker” (blue arrow) located on the corona of the glans penis of a 63-year-old Caucasian man

A 63-year-old Caucasian man, with a prior history of actinic keratoses treated with liquid nitrogen cryotherapy, presented for a total body skin check. He had no history of sexually transmitted infections. A cutaneous examination revealed a 2x2 millimeters purple papule on the corona of his penis (Figure 1). Further history elicited that the lesion was asymptomatic and had been present for 30 years. A correlation of the clinical presentation and lesion morphology established the diagnosis of penile angiokeratoma.

## Discussion

Angiokeratomas present as red, purple, blue and/or black, typically dome-shaped papules. They range from 0.5 to 5 millimeters in diameter. Five subtypes of angiokeratomas have been described: angiokeratoma circumscriptum, angiokeratoma of Fordyce, angiokeratoma of Mibelli, solitary angiokeratoma, and angiokeratoma corporis diffusum. There are several metabolic diseases in which angiokeratomas may present, including aspartylglucosaminuria, B-galactosidosis, B-mannosidosis, galactosialidosis, and Schindler/Kanzaki disease [17].

Angiokeratoma corpus diffusum is associated with a systemic disease such as Fabry’s disease or fucosidosis [9]. The proposed pathogenesis of angiokeratoma corporis diffusum in the setting of metabolic diseases is not completely clear; it is speculated that the accumulation of pathogenic metabolic substrates secondary to enzyme deficiencies within the cytoplasm of endothelial cells induces their apoptosis. This is followed by continuous reactive regeneration, leading to the weakening of capillary walls and the development of ectasia [13,17].

Angiokeratomas of Fordyce are most often observed on the scrotum or vulva but have also been noted on the glans penis and clitoris [1-3]. Angiokeratomas of the penis are rare vascular lesions that most commonly present as multiple lesions along the coronal rim of the glans penis [13]. Our patient presented with a solitary penile angiokeratoma; the morphologic features of the lesion were most suggestive of an angiokeratoma of Fordyce.

Twenty-one men with angiokeratoma of the glans penis have been described, including our patient (Table 1) [4-16]. The age of diagnosis of patients with angiokeratoma of the glans penis ranged from seven years to 80 years, with a median of 35 years. However, similar to our patient, the angiokeratomas may have been present for several years prior to the man seeking an evaluation of penile lesions.

**Table 1:** Reports of angiokeratoma of the glans penis C, case; CR, current report; DS, dorsal surface; Er:YAG, erbium-doped yttrium aluminum garnet; GP, glans penis; KTP, potassium titanyl phosphate; L, left; mm, millimeters; Nd:YAG, neodymium-doped yttrium aluminum garnet; nm, nanometers; NOS, not otherwise specified; NR, not reported; PDL, pulsed-dye-laser; R, right; Ref, reference; VS, ventral surface; y, years; &, and ^a^This is the age of diagnosis. For some of the patients, the initial presentation of the angiokeratoma was at a younger age. ^b^Also one lesion on corpus penis

C	Age^a^ (y)	Location	Size (mm)	Color	Treatment	Ref
1	7	Distal glans	4-5	Dark purple to black	595-nm PDL	[4]
2	10	Corona	2-4	Dark red to blue	532-nm Nd:YAG laser	[5]
3	14	DS & VS	2-4	Dark red-purple	None	[6]
4	26	Corona	2-4	Black	Electrocautery excision	[7]
5	26	GP: NOS	NR	NR	595-nm PDL and 1,064 nm Nd:YAG laser	[8]
6	28	GP: NOS^b^	NR	NR	Variable-pulse-duration PDL	[9]
7	28	GP: NOS	NR	NR	Variable-pulse-duration PDL	[9]
8	29	GP: NOS	NR	NR	Variable-pulse-duration PDL	[9]
9	31	GP: NOS	NR	NR	595-nm PDL and 1,064 nm Nd:YAG laser	[8]
10	35	GP: NOS	NR	NR	595-nm PDL and 1,064 nm Nd:YAG laser	[8]
11	35	GP: NOS	NR	NR	Variable-pulse-duration PDL	[9]
12	42	Corona	0.5-3	Blue-purple	None	[10]
13	43	Corona	2-4	Red-purple	Excisional biopsy	[11]
14	49	GP: NOS	NR	NR	Variable-pulse-duration PDL	[9]
15	52	Corona	1-2	Dark red to blue	2,940-nm Er:YAG laser and 532-nm KTP laser	[12]
16	58	GP: R side	1-2	Purple-black	Excisional biopsy	[13]
17	62	Distal corona	0.5-2	Red	None	[14]
18	63	Corona	2	Red-purple	None	CR
19	66	GP: L side	2-4	Red-purple	None	[11]
20	71	VS	1-4	Red-purple	Emollients	[15]
21	80	DS & VS	0.5-1	Purple	None	[16]

Most patients presented with multiple lesions. However, our patient is the second man to only have a solitary lesion on his glans penis. The other man was 58 years old when he had an excisional biopsy of the angiokeratoma on the right side of his glans penis [13].

The lesions ranged in color (red, blue, purple, and/or black) and in size (0.5 to 5 millimeters in diameter). The majority of the patients’ angiokeratomas were asymptomatic. Two men had minor associated bleeding [4-5].

The most common lesion site was the corona of the glans penis (33%, seven of 21 men), similar to our patient. However, the angiokeratomas were also found on the ventral and dorsal surfaces of the glans penis (9.5%, two of 21 men) or the right (4.8%, one of 21 men) or left (4.8 percent, one of 21 men) side of the glans penis. One boy’s angiokeratomas were noted on the distal glans. In addition to a lesion on the glans penis, one man also had a concurrent angiokeratoma on the corpus penis [9]. The clinical differential diagnosis of angiokeratoma of the glans penis is listed in Table 2.

**Table 2:** Clinical differential diagnosis of angiokeratoma cm, centimeters; mm, millimeters; AIDS: acquired immunodeficiency syndrome

Lesion	Distinguishing features
Basal cell carcinoma	Superficial type appears as dry, flat papules or plaques, with a raised border. The nodular type is pearly and lucent, with small telangiectasis and a rolled edge. Crusting and bleeding with minor trauma may occur. A non-healing ulcer may form. The pigmented type has border irregularities and possible color variegation. They are found in sun-exposed areas. Telangiectases, umbilication, and ulceration are possible on dermoscopy.
Capillary aneurysm	Flesh-colored solitary lesions resemble an intradermal nevus and are surrounded by erythema. They may suddenly grow larger and darker (blue-black) due to thrombosis.
Cherry hemangioma	Round, slightly elevated ruby-red papules are 0.5-6 mm in diameter. They increase in number with age and are found most often on the trunk. Early lesions mimic petechiae.
Condyloma acuminata	Lobulated papules are 2-5 mm and often multifocal. They are found in sites traumatized by sexual intercourse. Cauliflower-like masses may develop in moist, occluded areas. They are gray, pale yellow, or pink.
Dermato-fibroma	A single, round/ovoid, firm papule or nodule, which is 0.5-1 cm in diameter. They are red-brown or sometimes yellowish in hue. They may be elevated or slightly depressed, are usually on extremities, and produce a positive “dimple sign.” They can be initiated by bites or blunt trauma.
Kaposi’s sarcoma	Red, violaceous, or bluish-black macules and patches coalesce into rubbery plaques. They appear most often on the toes or soles and can extend to other areas. Oral and other mucosal tumors may occur. AIDS-associated cases present with numerous, symmetric, widespread lesions starting as macules and progressing to tumors or nodules.
Melanoma	Variants can be pedunculated, polypoid, amelanotic, or hyperkeratotic. They are often solitary, asymmetric, irregularly bordered lesions that evolve subacutely. Color tends to be variegated with a diameter >6 mm.
Petechial angioma	Multiple flat, irregularly round or angular, bright red lesions are 0.2-3 mm in size. They occur mostly on the trunk and upper extremities and partially blanch with diascopy.
Pyogenic granuloma	Small, eruptive, usually solitary, sessile or pedunculated, friable papule that occurs most often on an exposed surface or on gingiva in pregnant women. They bleed easily with trauma and recur if cut superficially.
Seborrheic keratosis	Oval, slightly raised, tan or light-brown to black, sharply demarcated papules or plaques are rarely more than 3 cm in diameter. They appear “stuck on” the skin. They occur mostly on the chest and back with occasional genital lesions. The crumbly surface, when removed, reveals a raw and moist base. They can be associated with itching.
Spitz/Reed nevus	Pink, smooth-surfaced, raised, round, firm papules are most often solitary but are infrequently agminate or disseminated. They can be pigmented or blue-black in color. A starburst pattern can be seen on dermoscopy. They typically arise in children.
Squamous cell carcinoma	Superficial, discrete lesion arises from an indurated, rounded, elevated base. They are dull red in color and contain telangiectases. Over months, they become larger, nodular, and ulcerated. The ulcer is initially hidden by a crust. New masses within scars or chronic ulcers, or an immunosuppression history, is suggestive. The skin shows signs of chronic sun exposure. Scale, crystalline structures, and keratin pearls are seen on dermoscopy.
Verruca vulgaris	Elevated, rounded papules with a rough, grayish surface are variable in size (1 mm to greater than 1 cm). They appear often as several scattered lesions. Immunosuppression is a risk factor. Thrombosed capillaries and dermatoglyphics are visible on dermoscopy.

Dermoscopy is a useful tool in differentiating between angiokeratoma and other lesions such as melanoma. Angiokeratoma is characterized by a lacunar or multicomponent pattern, with large, well-delimited, round to oval, red to black areas. There is also a whitish veil attributed to the acanthotic and hyperkeratotic epidermis [18].

The pathology features of penile angiokeratoma are ectatic blood vessels in the upper dermis in addition to acanthosis and/or hyperkeratosis. In some patients, the blood vessels were thrombotic. An “epidermal collarette” – in which vascular spaces were bordered by an extension of the epidermis into the dermis – could be identified [15].

The pathogenesis of genital angiokeratomas remains to be definitively established. Among the proposed hypotheses are congenital defects in venule walls and acquired injury to vessel walls due to trauma or long-standing venous hypertension [13]. Our patient did not have any history of trauma to the area or other lesions suggestive of a systemic disease.

The treatment of genital angiokeratomas may consist of observation since they are benign and asymptomatic. However, for lesions that are cosmetically undesirable or symptomatic, multiple potential treatment modalities are available. The various therapeutic methods include cryotherapy, electrocoagulation, laser, sclerotherapy, and surgical excision [4].

Nearly one-third of the patients with penile angiokeratomas (six men) did not require any treatment. Three men (14 percent) had their lesion excised. The excision either occurred during the biopsy (two men) or using electrocautery (one man).

Eleven men (52 percent) had their angiokeratomas treated with laser. One of the men had numerous lesions coalescing into a violaceous crusted plaque that was treated with a 595-nm pulsed dye laser, resulting in complete clinical resolution [4]. Other lasers that have been successfully used have included a 532-nm neodymium:YAG laser, an erbium:YAG laser, and a 532-nm KTP laser [5,12,19].

## Conclusions

Angiokeratomas of the penis are uncommon benign vascular tumors that most typically present as multiple lesions along the coronal rim of the glans penis. However, albeit less often, they can occur as a solitary lesion. Penile angiokeratomas range in size from 0.5 to 5 millimeters. The management of angiokeratomas of the penis is often observation; yet, laser treatment or excision have been successfully employed to remove the lesions. We suggest that this unique variant of genital angiokeratoma on the glans penis be referred to as a “peaker” (penile angiokeratoma).
